# 한국의 초산모들의 산후 사회적 지원 경험: 현상학적 연구

**DOI:** 10.4069/whn.2024.06.12

**Published:** 2024-06-28

**Authors:** Eunjoo Lee, Kyongsuk Hong

**Affiliations:** Department of Nursing, Kyungnam University, Changwon, Korea; 경남대학교 간호학과

**Keywords:** Mothers, Postnatal care, Qualitative research, Social support

## Abstract

**Purpose:**

Social support is essential for postpartum well-being, but little is known about the postpnatal social support preferred by primiparous women. This study aimed to comprehensively understand and describe the meaning of postnatal social support experience in primiparous women.

**Methods:**

The participants were seven primiparous women who were within 1 year after childbirth, recruited through purposive and snowball sampling from an online parenting community. The data were collected through in-depth interviews from November 14 to 28, 2022. Participants were interviewed face-to-face or via phone or online platform, according to their choice. Colaizzi’s phenomenological qualitative research method was applied to analyze the meaning of the participants’ experience.

**Results:**

Five theme clusters and fourteen themes were identified from the data. The five theme clusters are as follows: (1) Shortcomings of the childbirth and postpartum care system I learned through my experience; (2) Government policies focusing on childbirth and child-rearing rather than postpartum recovery; (3) Driving force of postpartum recovery: Shared childbirth process; (4) Childcare on my own; and (5) Conflicted between being a stay-at-home mom and a working mom under inadequate maternity protection policies.

**Conclusion:**

Despite postpartum support from the government that was perceived as inadequate, first-time mothers regained confidence and motivation for parenting with the help of family, peers, and social networks. First-time mothers need support from professionals and reliable online communities for postpartum recovery and parenting.

## Introduction

산후 기간은 여성과 가족에게 신체적, 심리적, 사회적으로 적응이 필요한 기간이며, 산모와 영아의 삶에 중요한 단계이자 모성 역할 획득과 정체성이 확립되는 모성 전환기에 해당된다[1]. 이 시기는 산모와 신생아 건강 관리의 측면에서 매우 중요하며, 질병 위험이 높지만 적절한 보살핌과 지원이 제공된다면 장기적으로 건강을 개선할 수 있는 가능성 또한 매우 높다[1]. 또한 산모는 이 기간에 새로운 가족관계와 모성 역할에 적응해야 하나, 일부 산모들은 적응 과정 중 불안이나 스트레스와 같은 산후 고통이나 정신 질환에 이환될 수 있어 이에 대한 적절한 관리가 요구된다[2].

사회적 지지는 주변 사람으로부터 받는 다양한 형태의 도움으로, 정서적(감정, 친밀감, 공감, 존경, 신뢰), 도구적(유형의 상품, 서비스), 평가적(긍정적 자기평가, 피드백) 및 정보적 지지(정보, 충고)의 4가지 유형을 포함한다[3]. 사회적 지지는 전반적인 웰빙(well-being)에 대한 부정적인 영향을 완화하는데, 개인이 스트레스에 직면했을 때 신체적, 정신적 및 감정적 웰빙을 보호하는 매우 중요한 요소로 알려져 있다[4]. 특히 개인이 사회적 지지를 받는다고 인식하는 것만으로도 삶의 만족도와 주관적 행복감이 높아질 수 있다고 보고된다[5]. 산후 사회적 지지는 출산 후 여성의 회복과 적응을 돕는 포괄적인 지원을 의미하며, 가족, 친구, 이웃, 지역사회 봉사자, 건강전문가 등 다양한 대상으로부터 받는 지지가 포함된다[2]. 산모가 산후에 충분한 사회적 지지를 받을 경우 산모의 신체적 및 정신적 건강이 증진되며, 삶의 만족도가 높아진다[2]. 또한 양육 자신감과 기술이 향상되며, 부모 역할에 대한 스트레스에 잘 대처하도록 하여 성공적인 부모 역할로의 전환이 가능하다[6].

이와 같이 산후 사회적 지지는 출산 후 여성의 신체적 및 정신적 건강에 중요한 역할을 하지만, 종종 과소평가되거나 충분히 제공되지 못하는 경우가 많은 것으로 알려져 있다[7]. 산후관리 전담 클리닉의 부족과 산모 대상 정규 교육 프로그램의 부재는 산모에게 필요한 정보, 교육, 지원 및 서비스 제공에 어려움을 초래하는 것으로 보고된다[8]. 또한 건강관리자가 제공하는 비판적 조언은 산모의 자신감을 떨어뜨리고 무력감을 느끼게 하며, 출처별 일관성 없는 정보들은 산모가 신뢰할 수 있는 정보를 찾기 어렵게 만들 수 있다[8]. 그리고 산모가 원하는 사회적 지지의 유형과 제공되는 사회적 지지의 유형이 다른 경우, 모성으로서의 역할 수행에 대해 부정적인 인식을 심어주며, 지지 인력과의 미흡한 상호작용이나 지원 환경에 대한 스트레스를 증가시킬 수 있다[6]. 부적절하게 구조화된 산후 사회적 지지는 부모로의 성공적인 전환에 불안정을 초래할 수 있으며[9], 불안 및 산후우울과 같은 정신질환[10], 경제적 어려움, 아기와의 애착 형성의 어려움 등 다양한 부정적 결과로 이어질 수 있다[9]. 따라서 산후 사회적 지지는 산모에게 맞춤화되어야 하며[4], 산모의 필요를 충족시켜야 한다[10].

경산모와 비교하여 초산모는 육아와 엄마 역할에 대해 더 높은 수준의 부담과 불안을 경험하는 것으로 보고된다[9]. 초산모는 수유, 아기 재우기, 아기 목욕 등 처음 경험하게 되는 육아 기술에 직면하게 되며, 특히 모유 수유를 가장 힘들어하는 것으로 나타났다[9]. 즉, 출산 경험이 없는 초산모는 출산 후 신체적, 정신적 변화에 대한 이해와 대처, 부모 역할에 대한 준비가 부족하기 때문에 산후 사회적 지지가 더욱 필요하다.

초산모의 산후 사회적 지지와 관련된 선행 연구를 살펴보면, 국외의 경우 산후 초기 초혼 부모의 사회적 지지 욕구[10], 산후 사회적 지지 경험[8], 사회적 지지에 대한 인식[2], 동료 지원[11] 등이 있었다. 국내 질적연구로는 초산모의 산후조리원 이용 경험 분석[12], 유도분만 경험[13], 임신과 출산 경험[14], 제왕절개 분만 경험[9], 산후조리원 경험[15] 등이 있었다. 이처럼 초산모의 사회적 지지 경험에 대한 연구가 국외에서는 활발히 이루어지고 있지만 국내에서는 출산과 조리원 경험에만 초점을 맞추고 있으며, 산후 사회적 지지 경험을 다룬 연구는 매우 부족하다. 따라서 초산모의 산후 사회적 지지에 대한 요구를 확인하여 적합한 지지를 제공하기 위해서는 이에 대한 심층적 연구가 매우 필요한 상황이다.

이에 본 연구에서는 첫 출산 후 산모가 받은 다양한 사회적 지지 경험이 어떠한지, 어떤 지원이 효과적이며 어떤 부분이 개선이 필요한지에 대해 탐구하고자 한다. 이를 위해 Colaizzi [14]의 현상학적 분석 방법을 사용하여 산후 사회적 지지에 대한 초산모의 공통적인 경험을 깊이 있게 이해하고자 한다.

본 연구의 목적은 초산모의 산후 사회적 지지 경험의 본질을 심층적으로 이해하는 것이며, 구체적으로 초산모가 산후에 경험하는 사회적 지지의 본질적 구조를 탐색하는 것이다. 이에 따른 본 연구의 질문은 “초산모의 산후 사회적 지지 경험은 무엇인가?”이다.

## Methods

**Ethics statement:** This study was approved by the Institutional Review Board of K University (IRB-1040460-A-2020-039). Informed consent was obtained from the participants.

### 연구 설계

본 연구는 초산모의 산후 사회적 지지 경험의 본질을 심층적으로 이해하기 위해 Colaizzi [14]의 현상학적 방법을 적용한 질적연구이다. 본 연구는 COREQ (Consolidated Criteria for Reporting Qualitative Research) 가이드라인에 따라 기술하였다[16].

### 연구 대상

본 연구 참여자는 첫 자녀를 출산한 산모로, 연구 주제에 대해 풍부한 경험을 가지고 있으며 적절한 정보를 제공하고 명확한 설명이 가능하다고 판단되는 대상으로 선정하였다. 구체적 선정기준은 (1) 만 20세 이상, (2) 출산 후 1년 이내, (3) 연구의 목적을 이해하고 연구 참여에 자발적으로 동의한 자이며, 자녀를 출산한 경험이 있는 경산모는 제외하였다. 출산 후 1년 이내의 기간을 선정한 이유는 이 시기가 출산 후 산모의 적응 과정에서 다양한 차원의 지지나 지원이 필요하며, 산모의 요구가 변화하는 기간이기 때문이다[17].

창원시 소재 한 인터넷 커뮤니티(사랑방)에 모집 공고문을 게시하여 연구 참여에 관심이 있는 경우 연구 책임자에게 유선으로 연락을 취하도록 하였다. 연구 책임자는 연구의 목적, 방법 등에 관해 설명하고 연구 참여에 자발적으로 동의할 경우 연구 참여자로 선정하였으며, 먼저 선정된 대상자들에게 친분이 있는 대상자를 소개받은 눈덩이 표집법(snowball sampling)을 활용하여 이차적으로 대상자를 모집하였다. 본 연구 참여자 수는 더 이상 새로운 진술이 나타나지 않아 자료가 포화된 시점을 기준으로 총 7명을 최종 선정하였다.

### 자료 수집

자료는 2022년 11월 14일부터 11월 28일까지 심층 면담을 통해 수집하였다. 참여자가 원하는 바에 따라 1:1 대면 혹은 비대면으로 면담을 실시하였고, 대면 심층 면담의 경우 설명 즉시 서면동의서를 받았으며, 비대면(줌, zoom) 심층 면담일 경우 설명 이후 메일이나 카톡으로 동의서 파일을 보내고 연구 참여자의 자필 서명 후 스캔 파일을 받았다.

면담을 시작하기 전 출산 이후의 일상적 내용을 중심으로 대화를 나누면서 참여자와 어색한 분위기를 완화했다. 참여자에게 “산후에 주변으로부터 어떠한 종류의 사회적 지지를 받으셨는지 말씀해주세요”와 같이 포괄적인 내용을 포함한 개방형 질문을 먼저 시작하였다. 부가적 질문으로는 “출산 이후 적응해 나가는 과정에서 어떠한 도움을 받으셨나요? 떠오르는 것들을 모두 이야기해 주시겠어요?”, “출산 이후 가족이나 주변 사람들로부터 어떠한 지지를 받으셨는지 이야기해 주시겠어요?”, “출산 이후 도움이 된 사회적 지원이나 환경은 무엇인가요?”, “산후에 산모에게 가장 필요한 도움이 무엇이라고 생각하시나요?”, “귀하께서 받은 도움 중 가장 좋은 것은 무엇인가요? 가장 아쉬운 것은 무엇인가요?”, “출산 이후 회복하는 데 힘든 점은 어떠한 것이 있었나요?” 등이었다. 1차 면담은 대상자별로 1회 실시하였고, 평균 50분, 최소 40분에서 최대 1시간 소요되었다. 4명의 대상자에게는 추가적인 자료가 필요하여 전화, 문자, 줌 화상회의 등을 활용하여 1–2회의 추가 면담을 실시하였으며, 평균 15분 내외가 소요되었다. 참여자에게 동의를 얻어 면담 내용을 녹음하고, 면담 당시의 느낌을 메모하였다. 면담이 끝난 직후 연구 책임자는 녹음 파일을 들으면서 직접 필사하고, 필사 내용을 바탕으로 분석하였다.

### 자료 분석 방법

본 연구는 Colazzi [14]가 제시한 현상학적 방법으로 분석하였다. 1단계는 녹음된 면담 내용을 반복해서 들으면서 연구자가 참여자의 언어로 필사한 후, 반복적으로 필사본을 읽으면서 참여자의 산후 지지에 대한 전체적인 의미를 파악하고자 노력하였다. 2단계는 필사한 내용에서 산후 사회적 지지와 관련하여 주요 내용에 밑줄을 그으며 의미있는 진술을 도출하였다. 3단계는 참여자들에게 의미있는 진술을 토대로 더 추상적이고 일반적인 형태로 주제를 분류하고 연구자의 언어로 표현하였다. 4단계는 도출된 의미 중 유사한 주제 묶음을 범주화하였다. 5단계는 주제 묶음들이 나타나는 현상에 초점을 맞추어 포괄적으로 기술하고, 핵심 범주와 본질적 구조를 도출하였다. 6단계는 참여자 2인에게 분석내용을 제시하여 그들의 경험과 일치하는지 확인하고 질적연구 전문가 2인과 본질적 구조의 타당성을 논의하였다. 질적분석 과정에서 목적적 표집을 통해 연구 질문이나 연구 목적을 고려하여 연구 질문에 대해 미리 제시하고 연구 대상자의 조건에 부합하는 대상자 중 본인이 잘 응답할 수 있다고 표현하는 대상자를 새로운 면담 대상으로 선정하였고, 수집된 자료를 분석하면서 연구의 목적이나 연구 질문에 충분히 대답할 수 있는 자료가 수집될 때까지 지속적으로 자료의 포화도를 검토하였다.

### 연구의 엄격성 확보 및 연구자 준비

#### 연구의 엄격성 확보

연구의 질을 검증하고 타당성과 신뢰성을 높이기 위해 Lincoln과 Guba [18]가 제시한 기준을 적용하였다. 사실적 가치를 확보하기 위해 녹음한 자료는 연구 책임자가 면담 24시간 이내에 듣고 필사하였으며, 녹음 자료를 2회 이상 반복해서 들으며 필사 자료와 비교하는 과정을 거치고 필사 내용 중 의문이 있으면 참여자에게 전화나 카카오톡으로 문의 후 추가 면담(약 15분 소요)을 진행하였다. 적용성을 확보하기 위해 산후 사회적 지지에 대한 경험을 충분히 표현할 수 있는 대상자로 선정하였다. 또한 연구 결과를 다른 참여자에게 보내어 참여자의 경험이 서로 일치하는지 확인하였다. 일관성을 확보하기 위해 자료 분석 시 Colaizzi [14]가 제시한 분석 방법에 충실하였으며, 연구 질문을 지속적으로 떠올리며 자료 수집 및 자료 분석을 실시하고, 질적연구 경험이 있는 간호학 교수 2인에게 개념과 범주에 대한 피드백을 받았다. 중립성을 확보하기 위해 연구자들은 출산 후 엄마로서 가지고 있는 이해와 편견을 공유하고 편향된 시각을 최대한 배제하도록 논의하였고, 면담 시 연구자의 개입을 최소화하였다.

#### 연구자 준비

본 연구에서 제1 저자는 질적연구 특강이나 워크숍 등에 참여하여 질적연구 설계, 질적내용 분석, 현상학, 근거 이론 등 질적연구 방법론을 꾸준히 익혀왔다. 현상학적 연구 방법론을 이용한 질적연구를 학회지에 여러 편 게재한 경험이 있으며, 주산기 여성의 우울에 관한 연구를 10년간 수행해 오고 있다. 교신저자는 질적연구를 수행하는 학회에 소속되어 질적연구 방법론과 관련된 특강과 워크숍을 꾸준히 참여하여 왔으며, 현상학적 연구 방법을 활용하여 학위 논문과 질적연구를 수행한 바 있다.

## Results

연구 참여자는 총 7명이었으며 연령은 평균 37.4세였다. 분만 형태는 제왕절개가 4명(57.1%), 질식분만이 3명(42.9%)이었으며, 여아를 출산한 경우가 4명(57.1%)이었고, 출산 후 자녀의 건강 상태는 7명(100%) 모두 양호하였다. 출산 후 기간은 평균 4.6개월이었으며, 주 양육자는 모두 산모 본인이었다. 주관적 경제적 수준은 좋음 1명(14.3%), 나쁨 6명(85.7%)이며, 고용 형태는 육아휴직이 4명(57.1%), 근무 중이 1명(14.3%), 무직이 2명(28.6%)이었다(Table 1).

본 연구에서 7명의 참여자로부터 얻은 초산모의 산후 사회적 지지 경험의 의미를 분석한 결과 28개의 의미 단위, 13개의 주제, 5개의 주제 묶음이 도출되었다(Table 2).

### 주제 묶음 1. 내가 경험해 보니 알게 된 출산 및 산후조리 시스템의 아쉬움

이 주제 묶음은 ‘모성의 역할 교육은 배제된 병원과 산후조리원’, ‘개별 전문성은 다르지만 그래도 도움이 된 산후 도우미’ 주제가 포함되었다. 참여자들은 출산 이후에 산후조리를 경험하며 본인에게 나은 산후조리 방법에 대해 알게 되었다. 다양한 산후조리 방법을 선택할 수 있지만, 공통적으로 병원, 산후조리원, 그리고 (병원 퇴원 및 산후조리원 퇴소 후) 집에서 받는 산후 도우미 서비스를 이용하는 일련의 과정을 거쳤으며, 이를 경험하면서 산후 회복과 아기 양육의 두 마리 토끼를 다 잡고 싶었지만 모성의 역할은 배제되어 아쉬움이 남았다고 하였다.

#### 주제 1.1. 모성의 역할 교육은 배제된 병원과 산후조리원

이 주제에는 ‘산후 의학적 처치에 집중하는 병원 입원 기간’, ‘산모의 신체적 회복에만 초점이 맞추어진 산후조리원’이 포함되었다. 참여자들은 출산 후 병원에서 대부분 기본적인 자궁수축 관리, 상처 관리 등의 산후 신체적 처치만 받았으며, 바빠 보이는 의료진에게 궁금한 아기 보살핌에 대한 자문을 받긴 어려웠다. 또한 병원 퇴원 이후 입소한 산후조리원에서는 아기를 봐주고 모든 것을 돌봐주는 덕분에 신체 회복은 수월히 이루어졌으나 실질적으로 아이를 어떻게 씻기고, 분유와 모유를 어떻게 먹이는지 등의 교육은 판촉 프로그램이나 브로슈어를 통해서 간접적으로 이루어졌다.

“대학 병원이다 보니 사실 그렇게 막 감정적으로 케어를 받거나 이런 거는 사실 크게 없었어요. 진짜 정말 기본적인 케어들만 받았고 이렇게 아이에 대해서 어떻게 하고 이런 거에 대해서는 따로 듣거나 지지받거나 그런 건 없었습니다.” (대상자 1)

“아이를 맡기고 내 몸은 쉴 수 있어서 산후 신체적 회복은 좀 된 것 같은데, 실질적으로 조리원에서는 애 마사지시키고, 그것도 사실 애를 데리고 실습을 해본 게 아니라 프린트물 같은 거 받으니까, 저는 사실 모르겠더라고요. 결국 마음대로 하게 되고... 진짜 초산모 입장에서는 아기 목을 어떻게 잡는지도 전혀 모르니까, 그래서 아기 목이 꺾이고 이런 일이 굉장히 많잖아요. (간단한 내용이 적힌) 프린트물만 주고, 이렇게 하면 된다고 설명하는 게 다니까 그렇게 도움이 되는지 모르겠어요. 그리고 아기 양육할 때 필요한 프로그램이라고 해서 짜여진 프로그램도 수유 방법은 분유 회사에서, 아이 마사지 방법은 베이비 오일 회사에서... 거의 제품 판촉을 위한 거라 교육 효과는 없었어요.” (대상자 2)

#### 주제 1.2. 개별 전문성은 다르지만 그래도 도움이 된 산후 도우미

이 주제에는 ‘내 몸도 쉬어 가며 하나씩 배울 수 있음’, ‘산후 도우미의 전문성은 경우에 따라 다름'이 포함되었다. 참여자들은 입원 혹은 조리원 생활 후 집으로 돌아와서 정부에서 지원해 주는 산후 도우미를 짧게는 1주, 길게는 2–3주까지 활용했다. 정부에서 지원하는 산후 도우미의 전문성에 따라 도움받은 내용은 상이했으나, 힘들 때 내 몸도 쉬어 가며 내 아이만 데리고 내가 궁금한 점을 하나씩 해결해 나가는 부분이 참여자에게 도움이 되었다. 다만, 산후도우미의 전문성은 아쉬운 점이었다.

“집에서 적응해야 되는 시간이 더 많잖아요. 근데 거기 이제 거기서는 모든 걸 다 해주니까 있을 때는 편하지만 나와서 이제 조금 부담스러운 게 있는데, 그 도우미를 차라리 한 2주 정도 더 하면 제가 이제 몸도 조금 더 편하기도 하고 적응도 조금 더 빨리 할 수 있을 것 같기도 해요. 어쨌건 제가 해야 하니까요.” (대상자 3)

“조리원 동기들은 산후 도우미에게 많은 도움을 받았다고 하던데... 저는 별로였어요. 정부에서 일괄적으로 다수의 사람들에게 지원하는 거면 그 사람들도 교육을 다 받고 왔을 건데 제가 만난 산후 도우미는 전문적 지식 없이 일하는 느낌이 좀 너무 좀 그렇더라고요. 정부에서 하는 거면 교육을 다 받지 않나?” (대상자 5)

### 주제 묶음 2. 산후 회복보다는 아기의 출생 및 양육 중심의 정부제도

이 주제 묶음은 ‘모든 지원은 아기에게 맞춰짐’, ‘고맙지만 아쉬움이 남는 제도’, ‘누구도 관심 없는 출산 이후 변화된 내 몸’ 주제가 포함되었다. 모든 국가적 지원은 기본적으로 저출산 문제 해결을 위한 방편으로 출생에 초점이 맞추어져 있으며, 아이와 첫 만남 이용권(출생 축하), 임신ˑ출산 지원비(임신, 출산 진료비 지원) 등으로 구분되었다. 그렇기에 산후 회복을 위해 활용할 수 있는 자원은 제한적이었으며, 출산 이후 산모의 변화된 몸 상태에는 아무도 관심이 없었다.

#### 주제 2.1. 모든 지원은 아기에게 맞춰짐

이 주제에는 ‘아기만 대접받고 산모는 뒷전’, ‘정부 지원은 임신, 출산기간 동안 아이를 위해 모두 활용함’이 포함되었다. 참여자들은 정부의 지원들이 저출산 정책으로 출산 장려를 하기 위해 산모보다는 아기에 초점 맞추어져서 만들어져 산모는 뒷전으로 밀려난 느낌이라고 표현했다. 또한 실질적으로도 정부 지원을 산모의 회복보다는 아이의 임신과 출산 과정에서 병원비, 물품 구매 등으로 모두 활용했다고 하였다.

“아이를 중심으로 모든 게 이렇게 흘러가요. 000 엄마라고 생각하고 그렇게 대하네요. 정책도 사실 아기 임신하고 낳는 데 초점이 되어 있잖아요? 정부의 지원은 아이 낳고 기르는 데 초점이 맞추어져 있을 뿐, 저의 산후 회복과는 멀리 있는 거 같아요.” (대상자 2)

“사실 육아라는 게 현실이고 이제 쌍둥이 같은 경우에는 뭐든지 두 배로 그렇게 비용이 들어서 현금 지원으로 급한 병원비와 아기들한테 들어가는 비용은 다 쓴 것 같아요. 저한테는 최소한만 쓸 수밖에 없죠. 들어가는 돈이 많으니까요.” (대상자 1)

#### 주제 2.2. 고맙지만 아쉬움이 남는 제도

이 주제에는 ‘유용하게 사용한 다양한 현금, 현물성 지원’, ‘번거롭고 일원화되지 않은 지원 절차’, ‘산모 소재지 중심의 지원적 한계’가 포함되었다. 참여자들은 정부에서 지원하는 다양한 현금, 현물성 지원은 아주 유용했고 초기 경제적 부담을 크게 줄여 주었다고 했으며, 지원이 확대되었으면 좋겠다고 하였다. 또한 번거로운 지원 절차를 보완하고, 소득에 상관없이 기본적인 산후 서비스는 제공받았으면 좋겠다고 표현했다. 초산모의 경우 실제적으로 지지를 해줄 수 있는 가족 근처로 가서 아이를 출산하고 산후 조리를 시행하는 경우가 많으므로, 산모 소재지 중심의 지원 제도는 변화해야 할 것이라고 조언하기도 했다.

“올해부터 생겼던 현금 지원 같은 것들이 많이 도움이 됐어요. 그거 아직도 조금씩 쓰고 있는데, 목돈 들어가는 곳이 많아서 유용하게 쓰고 있어요.” (대상자 4)

“다 좋은데 받는 데 과정이 너무 복잡해요. 직접 출산하고 나서 그 돈을 받기 위해서 동사무소를 가야 되는데, 조리원 퇴원하고 나서 몸 아픈 것 때문에 안 가게 되고 그 서비스를 받기가 힘들고, 혜택을 받기 위해서 정보를 확인해야 되는 여기저기를 거쳐야 되고, 한 번에 정리해서 보기 쉽게 해두지 않으면 신청하기도 어렵게 되어 있어서 힘들었어요.” (대상자 1)

“어중간하게 소득이 높은 사람들은 더 많은 혜택이 있기를 원하는 게 아니라 그 혜택을 모든 사람이 받을 수 있게 해줬으면 좋겠다고 생각해요. 저소득층한테 기저귀 바우처 주고 이런 거는 인정해요. 그런 거 받고 싶지는 않아요. 산후 도우미라든가 그런 것들은 기본적으로 좀 해줬으면 좋겠어요.” (대상자 6)

“저는 이제 제가 집이 원래 기장이지만 친정이 있는 창원에 가서 출산을 해서 그런 사회적인 혜택을 사실 아무것도 받을 수가 없는 거예요. 지역이 다르면 신청이 안 되더라구요? 결국 제 사비를 주고 해야 되니까... 그래서 그런 시스템을 아무것도 이용 못 했거든요. (중략) 친정에 가서 자녀를 낳는 사람들도 많을 것 같은데... 같은 지역인데 동만 다르다 이런 거면 상관이 없지만... 완전 지역이 다르면 저처럼 전혀 그런 제도적인 혜택을 못 받는 사람들도 의외로 많지 않을까라는 그런 생각이 좀 들었어요.” (대상자 2)

#### 주제 2.3. ‘누구도 관심 없는 출산 이후 변화된 내 몸’

이 주제에는 ‘육아하느라 나조차도 내 몸에 신경 쓸 겨를이 없음’, ‘출산 후 망가진 신체 변화’, ‘신체 회복을 위한 제도나 교육이 필요함’이 포함되었다. 아기가 집으로 오면서 밥 먹을 시간도 없이 아이를 돌보는 초산모는 자신조차도 본인의 몸을 돌볼 수 있는 여건이 되지 않았다. 아기를 보는 사이 한 번씩 들여다보는 거울에서 임신과 출산으로 인해 변화된 전신의 모습으로 인해 자존감도 떨어졌고 움직일 때마다 느껴지는 관절의 통증들이 참여자들을 힘들게 했다. 참여자들은 이러한 신체적 회복을 누군가가 관심을 가지고 도와줬으면 하는 바람을 표현하였다.

“아이가 생활 패턴이 좀 생겨야 그래도 시간을 계획할 수 있는데, 100일까지는 사실 패턴이라는 게 없으니까, 아이 잘 때 자고, 일어날 때 일어나고, 정신없이 아이의 생활 패턴에 맞추다 보니 저조차도 제 몸에 신경 쓸 수가 없어요.” (대상자 2)

“아이를 보면서 너무 예쁘다가도 거울을 보면 내 모습에 자존감이 떨어져요. 살도 많이 찌고, 머리도 빠지고, 체형도 변화되고, 서글프네요. 그래서 남편 스킨십도 싫어요. 그리고 잠 못 자면서 하루 종일 아기를 안고 있으니까 손목 발목 다 나가니까. 그게 제일 힘든 부분이에요. 허리, 손목, 무릎 관절이 너무 아파요. 몸이 제일 힘든 것 같아요. 지금도.” (대상자 6)

“운동 재활 같은 게 있잖아요. 지역 보건소나 체육센터 같은 곳이랑 연계해서 가벼운 산후운동이나 이런 걸 도와주는 그런 프로그램 같은 거 있잖아요. 그런 거 좀 좋지 않을까? 그런 생각이 저는 좀 들더라고요. 왜냐하면 애를 낳고 고관절이 너무너무 아팠어요. 그게 산부인과에 가서 물어봐도 시간이 지나야만 한다고만 이야기를 해주는 거예요... 그럼 저는 이제 회복 방법을 모르잖아요. 어떻게 해야 되는지 어떻게 회복해야 하는지를 알려주는 사람이 없는 거에요...그냥 시간이 지나면 낫는다 이렇게 얘기를 하는데 그게 얼마나 시간이 지나야 되는지… 골반을 다시 맞추기 위해서 어떤 운동이나 어떤 움직임을 하는 게 도움이 되는지... 그런 정보가 아무것도 없고 알려주는 방법도 없고... 저는 그런 게 좀 아쉽더라고요.” (대상자 2)

### 주제 묶음 3. 산후 회복의 원동력: 함께 하는 출산

이 주제 묶음은 ‘가장 든든한 우군, 친정 식구들’, ‘나의 편, 남편’, ‘사회적으로 환영받는 나의 임신과 출산’ 주제가 포함되었다. 참여자들은 산후 회복에 있어 가장 중요한 원동력이 가족으로부터 시작되었으며, 그 중 친정 식구들과 남편이 가장 핵심이라고 했다. 또한 나의 임신과 출산을 기뻐해 주고, 지지해 주는 사회적인 분위기를 경험하며 산후 회복을 해 나갔다.

#### 주제 3.1. 가장 든든한 우군, 친정 식구들

이 주제에는 ‘친정 부모님에게 육아와 가사를 전적으로 의지함’, ‘내 상황을 잘 아는 친정 식구들로부터 받는 크고 작은 도움’이 포함되었다. 참여자들은 산후 회복기간 동안 친정 부모님에게 육아와 가사를 전적으로 의지하고, 친정 식구들로부터 받는 크고 작은 도움을 통해서 점점 회복해갔다. 친정 부모님의 도움을 받으며 비로소 부모의 역할에 대해서도 생각해보기도 하고, 부모의 마음을 다시 헤아려 보기도 했다.

“아이를 처음 낳은 후 친정 엄마가 오셔서 거의 한 달 넘게 몸조리 끝날 때까지 친정 엄마가 전적으로 아기를 봐줘가지고 한 달 동안 저는 거의 애 안 보고 그냥 산후조리만 했는데 그때 참 고마웠어요. 그때 아기 낳고 몸이 많이 힘들었거든요.” (대상자 7)

“저희 친정 부모님들이나 이제 친정 식구들은 출산하고 선물도 많이 주시고 애들도 많이 예뻐해 주시고 이제 관심 같은 것도 많이 주시고 이래서 항상 이제 정서적으로 지지받는 느낌이었어요. 육아 조언이나 물품도 많이 받았어요.” (대상자 1)

#### 주제 3.2. 나의 편, 남편

이 주제에는 ‘육아 고충을 공감하는 남편의 말이 가장 큰 위안’, ‘남편의 헌신에 따라 산후 회복 정도가 달라짐’이 포함되었다. 참여자들은 잠시라도 최선을 다해주는 남편의 모습과 산후 육아의 고충을 이해하고 격려해 주는 남편의 말이 가장 큰 위로가 되었으며, 정서적 지지의 핵심이라고 표현하기도 했다. 또한, 같이 있는 남편의 헌신에 따라 산후 회복은 크게 달라졌다.

“남편은 (해외 근무로) 떨어져 지내지만, ‘몸은 괜찮아? 좀 회복했어?’ 이렇게 물어봐 주고, 제가 젖몸살을 진짜 심하게 해서 단유를 일찍 했는데 그때도 남편은 되게 덤덤하게 그렇게 얘기를 하더라고요. 지금까지 애썼다고 그렇게까지 더 애쓸 필요없다고... 그때 남편이 이렇게 내 감정을 이해해 주는 게 되게 위안이 되는구나 그런 생각이 많이 들었죠.” (대상자 2)

“항상 고마운데, 근데 진짜 남편이 진짜 좀 잘 해줘요. 집안일도 젖병 씻고 이런 거는 당연한 거고, 집안일도 잘해주고, 퇴근하고 오면 보통 한 10시, 11시까지 아기 봐주고 해서 제가 그렇게 진짜로 크게 힘든 건 없어요. 남편 덕분에 많이 회복된 것 같아요.” (대상자 5).

#### 주제 3.3. 사회적으로 환영받는 나의 임신과 출산

이 주제에는 ‘사회에서 어른으로 인정받는 것 같은 내 모습’, ‘축하해주는 주변 사람들의 격려와 지지’가 포함되었다. 참여자들은 임신과 출산을 통해 새로운 가족 구성원이 생기는 기쁨도 컸지만 비로소 사회적으로 책임과 의무를 다하며 어른이 된 것 같다고 표현하였으며, 이것을 주변 사람들의 격려와 지지로 확인하였다.

“아이를 낳고 나니 너무 예쁘고 기뻤어요. 세상에서 가장 소중한 존재잖아요. 아이 낳기 전에는 몰랐던 새로운 세상이예요. 아이를 낳음으로 어른으로 인정받는 느낌이고 내가 이 사회에서 책임과 의무를 다한 것 같아요.” (대상자 2)

“(임신했을 때에는) 길거리를 다니면 어르신들이 귀한 아이라고 축하한다고 해주고, 어디를 가도 그랬던 것 같아요. 항상 보는 사람마다 축하한다고 해주고, 마치 내가 이제 사회에서 어른이 된 느낌도 들었고 뿌듯했어요.” (대상자 1)

### 주제 묶음 4. 육아 홀로서기

이 주제 묶음은 ‘출산 후 완전한 회복 전 홀로 육아 현장에 내던져짐’, ‘동병상련 육아 동지로부터 받는 양육 정보와 위로’, ‘ 신뢰할 수 있는 정보가 없어 항상 고민인 첫 아이 육아’ 주제가 포함되었다. 초산모는 완전하게 산후 회복도 하기 전에 육아의 실전 현장에 투입되고, 아이와 관련된 다양한 경험을 하며 엄마로 거듭나고 있었다. 육아의 정답을 찾기 위해서 다양한 정보원을 찾아보기도 하고 조언을 구해보기도 했다. 첫 아이 육아는 항상 고민스럽고 어려웠는데, 이때 같은 처지의 엄마들의 위로는 큰 힘이 되었다고 하였다.

#### 주제 4.1. 출산 후 완전한 회복 전 홀로 육아 현장에 내던져짐

이 주제에는 ‘처음 경험하는 집안일 폭탄과 육아 전쟁’, ‘공부를 해보아도 답 없는 육아’가 포함되었다. 참여자들은 산후 도우미 지원 기간이 끝난 후 본격적으로 홀로 육아와 생활 속으로 진입했다. 참여자들은 아이가 생기면서 늘어난 집안일과 육아의 쳇바퀴 속에서 모성으로 거듭나고 있었으며, 매일 육아를 공부하며 아이를 돌봤으나 이게 맞나 고민이 되었다.

“아이가 50일 전후가 되면 제가 완전히 회복되지 않은 상태에서 저 혼자 아이도 보고, 집안일도 하기 시작해요. 그런데 아이가 깨어 있으면 집안일이 안 되니까 애를 재우고 틈나는 대로 밀린 집안일을 해야 해요. 집안일은 항상 밀려 있는 상태죠. 남편이 많이 도와주는 편인데도 일단 애를 재우고 나서야 집안일을 시작을 할 수 있어요. 아이 옷 세탁이며 젖병 소독 등 할 일이 산더미인데, 애도 봐야 하고, 밥 먹고 세수할 시간도 없이 전쟁처럼 하루하루 살고 있습니다.” (대상자 2)

“먼저 육아를 경험했던 언니들이나 가족들, 아니면 이제 유튜브 같은 것을 보고 공부하며 정보를 얻고는 있는데 사실 그게 저희 아이들에 딱 맞는 정보가 아니다 보니 (제 나름대로 열심히 공부하면서 아이를 키우고 있는데) 정답이 없는 것 같아요.” (대상자 1)

#### 주제 4.2. 동병상련 육아 동지로부터 받는 양육 정보와 위로

이 주제에는 ‘육아 정보를 빠르게 얻을 수 있는 가장 큰 정보원’, ‘육아 동지만 이해하는 육아 고충’이 포함되었다. 참여자들이 아이를 양육할 때 큰 도움이 되었던 것은 비슷한 또래의 아이를 양육하는 육아 동지였다. 나 홀로 고민하던 모든 정보를 오픈 채팅방이나 인터넷 카페를 통하여 습득했고, 육아 고충도 같이 공유하며 위로받았다. 같은 시기에 또래의 아이를 키우는 것은 엄마들로 하여금 강력한 연대감을 느끼게 하였다.

“맘카페에 주제어로 검색해서 많이 보거나, 아니면 그냥 인터넷 검색창에 검색해 가지고 블로그 같은 것도 참고하고요. 지금 (맘카페에서 만난 비슷한 시기의 아이를 양육하는 엄마들의) 오픈 단톡방 있잖아요. (여기 모인 엄마들의 아이들이 우연찮게) 진짜 다 같은 달 아기들이니까 너무 좋은 거예요. 저도 정보를 그나마 많이 얻는 곳이 저희 단톡방이거든요.” (대상자 3)

“새벽에 내가 카톡을 올리면 누군가는 수유를 하면서 보고 있으니까 나와 같은 시간대에 같은 일을 하고 있는 사람이 누군가 있구나… 그러면서 서로 경험을 이야기하며 조언받는 거예요. 만나지 못해도… 한밤중에 혼자서 진짜 분유를 주면서 억울하고 속상할 때도 그 방(산후조리원 동기 단톡방)에서는 비슷한 처치의 엄마가 있으니… 하소연을 하면 누군가는 공감을 해 주니까.” (대상자 6)

#### 주제 4.3. 신뢰할 수 있는 정보가 없어 항상 고민인 첫 아이 육아

이 주제에는 ‘정보는 많으나 신뢰할 수 있는 정보는 찾기 힘듦’, ‘내 아이에게 맞는 맞춤형 육아 전문가의 도움이 필요함’이 포함되었다. 참여자들에게 첫 아이 육아는 어렵고 힘들었고, 다양한 정보들 속에서 신뢰할 수 있는 정보는 찾기 어려웠으며, 전문가마다 말하는 내용이 다 달라 고민스러웠다고 하였다. 또한 참여자들은 내 아이에게 맞는 발달 단계에 따라 맞춤형 육아 전문가의 도움이 필요했으며 항시 상담할 수 있는 창구가 있었으면 좋겠다고 표현했다.

“육아 전문가라고 나오는 분들마다 너무 의견이 달라요. 분유량도 누구는 먹일만큼 먹여라, 누구는 1,000 mL 넘기지 마라… 누구의 말이 맞는지도 모르겠고, 충분히 신뢰할 수 있는 전문가로부터 교육받을 수 있는 통로가 있으면 좋겠어요.” (대상자 5)

“첫 아이니까 정말 아무것도 몰랐어요. 진짜 그 전에 다 찾아보아도 모르는 게 워낙 많다 보니까, 좀 자유롭게 신청해서 볼 수 있게끔 하는 전문가가 하는 그런 교육이 있다면 좋을 것 같아요. 목욕하는 영상도 유튜브 찾아보면 정확하게 자세하게 나와 있는 건 없더라고요. 그냥 럭비공 끼듯이 옆에 아이를 끼워서 이렇게 씻으면 된다고만 되어 있어요. 저는 제 아이가 신생아 때가 아니라 100일이 됐을 때 200일이 됐을 때 300일이 됐을 때 점점 씻는 과정들이 달라지잖아요. 그런 것들에 대해 알고 싶은데, 주변에도 친구들은 너무 오래돼서 모르고, 그런 영상은 찾아도 잘 안 나오더라고요. 그래서 육아 전문가가 운영하는 우리 아이 연령대에 맞는 온라인 소통 창구 같은 게 있어도 좋을 것 같아요.” (대상자 4)

### 주제 묶음 5. 양육 상황을 불완전하게 커버하는 모성보호 제도 속에서 전업주부와 워킹맘을 놓고 갈등함

이 주제 묶음은 ‘출산 후 사회에서 점점 멀어져 감’, ‘사회로 복귀하려면 아이는 하나만 낳아야 할 것 같음’의 주제가 포함되었다. 참여자들은 출산 후 회복하고 아이를 키우는 사이, 사회에서 멀어져 가는 본인의 모습을 인지하게 된다. 또한 육아휴직이나 출산휴가, 유연근무제 같은 모성보호 제도들이 많이 생겼지만, 아이 양육 과정 중 생기는 문제를 온전히 해결할 수 없기에 전업주부와 워킹맘 중 양자택일해야 하는 상황이 나타나 갈등하게 된다. 참여자들은 아이를 더 낳아서 키우고 싶지만 다양한 상황 속에서 아이는 하나만으로 충분하다고 생각했다.

#### 주제 5.1. 출산 후 사회에서 점점 멀어져 감

이 주제에는 ‘출산 후 남편, 회사 동료와 이야기의 주제가 달라짐’, ‘아이를 두고 복귀할 수 있을까 고민함’으로 나타났다. 참여자들은 출산 후 아이를 양육하는 사이에 남편, 회사 동료들과 이야기의 주제가 달라졌음을 인지하고 사회에서 동떨어진 것 같은 본인의 모습을 발견했다. 그리고 육아를 하면서 자연스럽게 아이를 두고 직장으로 복귀할 수 있을까 고민하게 되었다. 모성보호 제도가 있지만 실제로 활용할 수 있는 부분은 제한적이라, 양육 보조자가 없는 경우 직장 복귀를 고민하게 되었다.

“남편이나 직장 동료들이랑 한 번씩 이야기하면 이야기 주제가 완전 달라요. 저는 아이와 관련된 얘기만 하고 있어요. 약간 사회에서 멀어지는 느낌이 듭니다.” (대상자 2)

“제가 지금 직장 들어간 것도 지금 1년밖에 안 됐거든요. 여기 온 지 이사 온 지 얼마 안 되어서 그때도 이제 적응 기간이었는데 적응 다 하니까 이제 또 육아휴직을 또 하게 되어 또 어떻게 또 적응을 해야 되나 싶어요. 아이를 보면 내려놔야 되나 싶기도 하고, 지금 적응과 육아 두 마리 토끼를 잡을 수 있을까 하는 그런 노파심이 있네요.” (참여자 5)

“막상 제가 그 현실에 닥치니까 정말 대한민국에서 출산한 여자들이 진짜 선택지가 다양하지가 않구나 라는 생각을 많이 했거든요. 그래서 이제 우리나라가 출산율이 낮은 이유가 있겠구나 라고 생각했고, 저는 육아하면서 전업주부 하면서 개인 커리어 포기하고 남편이 외벌이로 벌어주는 돈으로 이렇게 아등바등 사는 것과 애들 맡기면서 회사 다니는 거 이런 거 둘 중 하나 어쩔 수 없이 선택할 수밖에 없는 대한민국 현실에 남편이랑 많이 싸우기도 했고 내적으로도 갈등이 심하게 생겼어요.” (대상자 1)

#### 주제 5.2. 사회로 복귀하려면 아이는 하나만 낳아야 할 것 같음

이 주제에는 ‘출산 후 자연스럽게 나의 것은 내려놓음’, ‘나를 희생해야 하는 경험은 한 번으로 족함’으로 나타났다. 참여자들은 임신과 출산, 육아 과정을 경험하고 사랑스러운 아이를 보며 자연스럽게 나의 것을 내려놓을 수밖에 없는 현실을 인지했다. 동시에 나의 커리어를 위해 나를 희생하는 경험은 한 번으로 만족한다고 표현했다.

“원래 옷 가게를 하면서 수입이 괜찮았어요. 그런데 아이를 낳고 나서 이것저것 따지니까, 결국 제가 집에서 아이를 봐야 하는 상황이 되거든요. 물론 아이가 예쁘기도 하지만, 자연스럽게 양가 부모님들도 당연하게 남편의 직장이 중요하다고 하시니, 내가 이때까지 쌓아왔던 것은 자연스럽게 내려놓게 되었어요.” (대상자 5)

“원래는 남편과 둘이서만 살기로 했는데, 아이 하나는 있어야 하지 않나? 라고 생각해서 남편이랑 상의하고 계획해서 출산을 했어요. 그런데 아이를 낳고 나니 예쁘긴 하지만, 제가 어느 정도 연차가 되서 직장생활 하면서 커리어를 쌓아서 경제적으로도 여유가 있었고, 둘이서 취미활동도 같이하고 했었거든요. 지금도 직장 복귀가 언제 될지 모르는데... 주변에서 나이 한 살이라도 어릴 때 하나 더 낳으라고 하시지만, 직장 복귀를 생각했을 때 출산의 경험은 이번 한 번으로 충분합니다.” (대상자 2)

## Discussion

본 연구는 초산모의 산후 사회적 지지 경험의 본질을 알아봄으로써 출산 후 여성들이 필요로 하는 지지에 대한 이해와 요구를 탐색하기 위해 수행하였다. 본 연구 결과 초산모의 산후 사회적 지지 경험은 5개의 주제 묶음과 13개의 주제로 도출되었으므로, 이를 중심으로 논의하고자 한다.

첫 번째 주제 묶음은 ‘내가 경험해 보니 알게 된 출산 및 산후조리 시스템의 아쉬움’으로, 참여자들은 모유 수유와 신생아 관리와 같은 모성 역할에 전문성을 갖춘 인력의 도움이 필요했다. 초보 엄마들은 어머니로서의 경험이 처음이기 때문에 엄마 역할에 대한 두려움과 불안이 높으며, 산후 전문가로부터 더 많은 정보와 지원을 요구하게 된다[11]. 그러나 전문성을 갖추지 못한 인력은 산모와 신생아에게 적절한 돌봄을 제공할 수 없으며, 오히려 산모에게 육아 부담감과 스트레스를 유발할 수 있다[19]. 또한, 초산모는 산후 인력의 형식적인 태도가 산모의 신체적, 정서적 요구를 충족하기에 미흡하다고 느끼며, 자녀가 없는 의료 전문가의 학문적 지식보다는 어머니의 경험적 지식을 더 신뢰한다고 언급하였다[20]. 따라서 초산모의 모성 역할 획득을 돕기 위해서는 모유 수유나 신생아 관리에 전문성과 경험이 풍부한 산후지원 인력의 정착이 필요하며, 산후 도우미 교육 및 자격 기준이 일관성 있게 정립되어야 할 것이다.

두 번째 주제 묶음은 ‘산후 회복보다는 아기의 출생 및 양육 중심의 정부 제도’로, 국가 지원의 문제로 인하여 일부 참여자들은 산후지원 프로그램이나 서비스에 접근이 어렵거나 지원 대상에서 제외되었다. 2021년 지자체의 출산지원 예산은 전년 대비 24.3% 증가한 4,441억 원으로 해마다 증가하고 있으며, 임신과 출산뿐 아니라 결혼 전부터 다양한 출산지원을 포함하고 있다[21]. 이는 저출산 극복 정책의 일환으로, 산후 서비스 정책은 장기적인 육아 환경을 구축하기 위한 방향으로 전환되고 있는 추세이다[22]. 하지만 현재의 정책에도 불구하고 초산모들은 산후지원의 기간이 짧고 제공되는 지원 금액이 부족하다고 인식하였다[13]. 국외 선행 연구에서도 초산모들은 출산 후 국가로부터 충분한 지원을 받지 못하거나 다양한 장벽에 직면한다고 보고되며[2], 이는 지원 정책이 초산모의 실질적인 요구를 충족하지 못하거나 실행과 운영 과정에서 어려움이 있을 수 있음을 시사한다. 최근 늘어나는 고령 초산모들은 젊은 초산모보다 분만 후 회복에 더 많은 시간이 필요하며, 주변에 기대할 수 있는 지원책이 부족하기 때문에, 현실적인 정책에 대한 기대가 더 크다[13]. 첫 출산 연령이 증가하는 것이 사회적 추세이고 본 연구에서도 초산모의 대부분이 35세 이상이므로, 고령 임부의 산후 요구에 부응하기 위해 산후지원의 기간과 비용을 더욱 확장하고 조정해 나가야 할 필요가 있다고 판단된다. 또한, 산모의 연령과 회복 기간을 고려하여 일회성의 현금 지원보다는 장기간에 걸친 산모 회복을 고려한 효과적인 지원 서비스를 제공하여야 할 것이다[22].

한편, 산후지원은 태아 유형, 출산 순위, 소득 수준, 서비스 기간에 따라 차등 지급되고 지방자치단체마다 지원 조건이 달라지기 때문에, 초산모들이 제대로 지원을 받지 못하기도 하였다[23]. 또한 최근 출산 지원 정책 모니터링 보고서에서 산모들은 지원 서비스에 대한 정보 부재와 신청 및 자격 판정과 같은 행정 프로세스에 대한 불편함을 호소하는 것으로 나타나[23], 본 연구와 유사한 결과를 보였다. 국가와 지방자치단체는 산후지원의 기준과 절차, 부작용에 대해 충분한 검토를 진행하고, 정책 수립과 실행 단계에서 이러한 문제점들을 고려해야 할 것이다. 또한 지원 정책 서비스의 효율성을 향상하기 위해서는 정보 제공 및 신청 프로세스 간소화에 주력해야 하며, 산모의 실질적 요구를 반영하여 지원 정책의 사각지대가 발생하지 않도록 정책을 개선할 필요가 있다.

초산모들의 산후지원에 대한 좋은 인식은 출산 후 스트레스를 감소시키고, 산후 건강과 웰빙을 향상하는 데 도움이 된다[4]. 이는 산후 지지에 대한 긍정적 인식이 초산모의 산후 경험에서 부정적인 측면을 최소화하고 초산모의 산후적응과 회복을 촉진할 수 있음을 의미한다. 국가와 지방자치단체는 산후지원의 기준과 절차, 부작용에 대해 충분한 검토를 진행하고, 정책 수립과 실행 단계에서 이러한 문제점들을 고려해야 할 것이다.

세 번째 주제 묶음은 ‘산후 회복의 원동력: 함께 하는 출산’으로, 참여자들은 가족과 사회로부터 임신과 출산의 힘든 과정을 공유하고 도움을 받을 때 산후 회복과 엄마 역할에 대한 자신감을 키워갈 수 있었다. 초산모는 육아 부담이 높으므로, 남편, 친정과 시댁에서 육아에 대한 비용 지원을 비롯한 실질적인 도움이 필요하다[24]. 산모들은 산후조리가 남편을 포함한 가족이 함께 노력하는 과정이라고 인식하였으며[17], 초산모에게 친정엄마와 배우자는 핵심적인 지원자로 언급되어[2] 본 연구 결과와 유사했다. 초산모 부부는 아기가 생긴 이후 서로의 건강에 더 관심을 가지게 되고 가족에 대한 사랑이 더 넓어지는 것을 인식하였다[13]. 따라서, 남편을 비롯한 가족들은 육아 교육에 적극 참여하여 전문적인 육아 지식을 습득하고, 육아에 대한 역할 분담을 함께 계획하여 초산모를 지지해야 할 것이다. 또한 가족이 없거나 가까이 살지 못해 도움을 받기 어려운 초산모들을 위해 지역사회 차원의 지원 프로그램을 제공하여야 하며, 병원, 보건소 등 지역 자원들이 연계하여 지원체계를 강화해야 할 것이다.

네 번째 주제 묶음은 ‘육아 홀로서기’이다. 참여자들은 산후 회복이 되기도 전에 육아 현장에 내던져져 홀로 첫 아이 육아를 잘 해내기 위해 많은 정보를 찾게 되지만, 서로 다른 의견과 상충되는 정보들로 인해 혼란을 경험하였다. 임산부들은 출산과 관련된 정보의 출처로 인터넷을 활용하는 경우가 가장 많았지만, 과잉이거나 신뢰할 수 없는 점으로 인해 인터넷 정보를 선호하지 않게 되었다[25]. 그들은 일관되고 신뢰할 수 있는 정보를 필요로 하며, 전문가에 의해 통제된 정보를 얻길 원했다[22]. 초산모는 육아의 기본 요령이나 지식이 부족하기 때문에 전문가로부터 받는 상반된 견해를 접할 경우 정보에 대한 신뢰가 급격히 손상될 가능성이 높다[8]. 따라서 정부기관, 의료 전문가, 혹은 육아 관련 단체는 출산과 육아에 관한 정확하고 일관된 정보를 제공하여 초산모들이 필요한 정보를 손쉽게 얻고 활용할 수 있도록 도와야 할 것이다.

또한 초산모들은 또래 엄마들과 온라인 혹은 그룹 메시지를 사용하여 육아 문제를 해결하는 경향이 높다[8]. 그리고 육아 모임이나 소셜 네트워크 서비스(social network service, SNS)를 통해 유용한 육아 정보를 얻을 뿐 아니라 서로 교류하고 소통하며 심리적으로 지지하게 된다[20]. 코로나 19 팬데믹 이후 병원과 산후조리원 이용이 제한되면서 임신, 분만, 산후조리에 있어서 SNS나 비대면의 활용이 더 높아지게 되어[26], 이러한 경향은 더 심화되었다. 최근의 초보 엄마들은 자녀의 건강에 대한 부담이 높고 많은 의료 정보를 빠르게 획득하길 원하기 때문에, 비슷한 연령의 자녀를 둔 여성들과 경험을 실시간 공유하고, 자신의 행동이 정상인지 확인하여 필요한 정보를 얻고자 한다[19]. 그러나 McLeish 등[8]의 연구에서 일부 산모의 경우 자신을 자랑하거나 자신의 욕구를 충족하며, 그룹 내 다른 산모들과 경쟁감을 느끼기도 하기 때문에 동료에게 의지하는 것의 위험성을 언급하였다. 초산모의 모성 역할을 돕기 위해 실시간 문제 해결이 가능한 육아 관련 단체 대화방이 필요하며, 전문가에 의한 상호작용과 안전한 환경이 갖추어져야 할 것이다. 또한, 초산모들은 전문가나 주변 사람들로부터 비판적이거나 지시적인 대화보다는 동료 엄마들과 비슷한 경험을 함께 나누고 자신을 표현하길 원한다[2]. 남편이나 가족이 있지만 동료로부터의 지원을 구하는 것은 자신의 감정을 공유하고 공감하는 경청자이기 때문이라고 하여[23] 정서적 연결의 중요성을 강조하고 있다. 특히 초산모들은 새로운 엄마가 되는 경험을 공유하고 싶어 하며, 같은 일을 겪고 있는 비슷한 연령의 자녀를 둔 여성들과의 대화를 지원하고 공유하는 것은 육아로 인한 외로움과 고립감을 극복하는 데 도움이 될 수 있다[19].

초산모는 수술 부위 및 회음 절개 부위 통증, 모유 수유로 인한 유두 통증, 허리와 손목 통증 등의 신체적 고통과 심리적 문제를 경험하며, 자신이 겪고 있는 변화와 치유 과정에 대해 조언을 받길 원한다[27]. 그러나 벨기에에서 수행된 선행 연구에 의하면[19], 초산모의 대부분은 산후조리에 대처하기 위한 교육이나 지원 네트워크가 부족하다고 느끼는 것으로 나타나 본 연구 결과와 유사했다. 산모는 출산 후 회복이 덜 된 상태로 아이를 돌봐야 하는 어려움에 직면하며[13], 특히 초보 엄마의 경우 출산 후 체력이 많이 소모되고 자신의 요구와 엄마의 역할 사이에서 갈등을 경험하게 된다[15]. 출산 후 호르몬 변화 및 여러 요인으로 인하여 불안과 스트레스를 경험하며, 모성으로 전환하는 과정에서 더 나은 엄마가 되어야 한다는 죄책감과 압박감, 아기의 건강에 대한 두려움 등으로 심리적 고통이나 정신적 위험에 처하게 된다[2]. 출산 트라우마가 있을 경우 출산 후 웰빙에 지속적으로 부정적인 영향을 미치기도 한다[2]. 초산모는 분만과 출산에 대한 경험이 없기 때문에 출산 후 자신의 신체적, 심리적 변화와 효과적인 관리 방법에 대한 정보를 필요로 한다[8]. 그럼에도 임신 시 산부인과 검진이 정기적으로 중요시되는 반면, 출산 후에는 산모를 위한 의학적 검사가 부족하고, 산후 진료는 신생아를 중심으로 실시되어[19] 이러한 문제는 제도적으로 개선해야 할 것으로 판단된다. 그러므로 초산모가 경험하는 산후 고통을 이해하고, 초산모의 신체 회복 및 심리적 문제와 관련된 정보와 서비스 지원을 강화해야 할 것이다[2]. 초산모는 자신이 산후조리의 주체가 되길 원하며, 산후조리를 출산 후 산모의 권리로 인식하기 때문에[17], 산모 개개인의 산후 요구와 회복 정도가 존중되어야 한다. 따라서 가족과 전문가들은 산후 초산모의 산후 회복을 위해 적절한 시기와 유형에 맞도록 개별화된 사회적 지원을 제공해야 할 것이다.

다섯 번째 주제 묶음은 ‘양육 상황을 불완전하게 커버하는 모성보호 제도 속에서 전업주부와 워킹맘을 놓고 갈등함’으로, 참여자들은 엄마가 되면서 워킹맘과 전업주부 사이에서 갈등하며 선택의 기로에 놓이는 모습이 나타난다. 최근 출산에 대한 인식 연구에 따르면, 20–39세 미혼남녀의 이상적인 자녀관에서 여성의 경우 30.0%가 자녀가 없는 것, 남성은 20.0%가 자녀가 없는 것이 이상적이라고 응답했다. 또한, 자녀를 더 가지기 위한 전제 조건으로 여성들은 배우자의 적극적인 양육 및 가사 참여와 평등한 관계를 가장 중요한 요인으로 꼽았고, 아울러 여성이 계속 일할 수 있는 환경이 중요하다고 인식하고 있었다[28]. 일터에서 경험하는 모성은 페널티를 유발하는 것으로 인식되는 현재 상황에서 초산모들이 경험해 보지 않은 워킹맘의 생활과 전업주부 사이에서 갈등하고 있는 것으로 생각된다. 따라서 출산 후 여성의 자율적 의지에 따라 아이 양육과 본인의 커리어를 선택할 수 있는 사회적 분위기와 이를 지지해 줄 수 있는 제도가 조성되어야 할 것이다.

본 연구 결과, 국가의 산후지원에 대한 구조적인 문제들로 인해 초산모들이 지원 서비스에 접근하는 데 어려움을 겪었고, 신뢰할 수 없고 방대한 정보로 인해 육아 선택에 혼란을 경험하였다. 강압적이거나 자신과 맞지 않는 육아 조언보다는 자신의 육아를 존중하고 공감해주는 사람들과의 유대에서 심리적 위안을 얻었다. 특히 초산모는 모유 수유와 신생아 돌보기와 같은 모성 역할 획득에 있어서 가족보다는 전문성과 경험을 갖춘 산후 도우미와, 같은 시기에 출산 경험을 가진 육아 동지들을 의지했다. 인터넷 정보보다는 신뢰할 수 있는 육아 컨텐츠를 선호했으며, 실시간으로 운영되는 육아 단체 대화방에서 육아 문제를 해결하고 있었다. 출산이 초산모만의 사건이 아니라 가족, 동료, 사회 모두가 함께 기뻐하고 책임져야 하는 일로 인식하고 실질적인 도움을 받게 될 때, 산후 회복과 육아에 대한 자신감과 의지를 가지게 되었다.

본 연구는 연령이나 분만 방식을 제한하지 않았기 때문에 연구 참여자로부터 출산 후 사회적 지지에 대한 공통적인 경험을 이끌어내지 못한 부분이 있다. 그러나 본 연구는 초산모의 관점에서 산후 사회적 지지에 대한 경험을 탐색한 국내 최초의 연구로, 고령의 초산모가 증가한 현 시점에서 초산모의 산후 요구를 탐색하고, 향후 출산 서비스와 정책의 방향을 확인했다는 점에 의의가 있다.

향후 초산모의 산후 사회적 지지에 대한 요구가 국가의 산후지원 정책 수립과 실행과정의 모든 단계에서 반영되어야 할 것이다. 구체적으로 산후조리와 부모됨에 대한 기본 교육의 강화, 산모의 건강과 회복에 중점을 둔 서비스 제공, 지역과 소득에 제한 없는 공평한 지원, 산후 관련 전문인력 확충, 전문가가 제공하는 육아 컨텐츠와 실시간 육아 상담 서비스 제공 등에 대한 노력이 필요하다. 후속 연구에서는 이러한 대상자의 특성을 고려하여 자료를 수집하고, 본 연구 결과를 반영하여 초산모의 산후 사회적 지지를 향상하기 위한 서비스나 프로그램에 적용할 것을 제언한다.

## Figures and Tables

**Table 1.** General characteristics of participants

ID	Age (year)	Delivery method	Child’s sex	Child’s health	Main caregiver	Duration after childbirth (month)	Form of employment	Perceived family economic status
1	32	C/S	Male twins	Good	Self	3	Unemployed	Poor
2	35	C/S	Female	Good	Self	4	Parental leave	Poor
3	34	C/S	Male	Good	Self	4	Unemployed	Poor
4	43	Vaginal	Female	Good	Self	5	Parental leave	Poor
5	39	Vaginal	Female	Good	Self	4	Parental leave	Poor
6	43	C/S	Male	Good	Self	6	On duty	Good
7	36	Vaginal	Female	Good	Self	6	Parental leave	Poor

C/S: Cesarean section.

**Table 2.** Postpartum social support experiences in primiparous women

Theme clusters	Themes	Meaning units
Shortcomings of the childbirth and postpartum care system I learned through my experience	Hospitals and postpartum care centers lack maternal role education	- Hospital stay focused on medical treatment during the postpartum period
		- Postpartum care centers focus only on physical recovery of the mother
	Postpartum helpers are helpful despite varying levels of expertise	- I can rest and learn one thing at a time
		- The expertise of postpartum helpers varies case by case
Government policies focusing on childbirth and child-rearing rather than postpartum recovery	All support is focused on baby	- Only the baby receives attention while the mother is neglected
		- Government support is entirely used for the child during pregnancy and childbirth
	Appreciative yet finding the system lacking	- Various cash and in-kind supports were used effectively
		- Support procedures are cumbersome and not streamlined
		- Support limitations based on the mother’s location
	No one cares about the changes in my body after childbirth	- Busy with childcare, I don’t even have time to care for my own body
		- Physical deterioration after childbirth
		- Need for systems or education focused on physical recovery
Driving force of postpartum recovery: Shared childbirth process	Most reliable allies, my family of origin	- Relying entirely on my parents for childcare and housework
		- Receiving various forms of help from family members who know my situation well
	My supporter, my husband	- My husband’s empathetic words about the struggles of childcare provide the greatest comfort
		- The degree of postpartum recovery varies depending on my husband’s dedication
		- I feel like an adult in society
		- Encouragement and support from people around me who celebrate with me
	My pregnancy and childbirth welcomed by society	
Childcare on my own	Thrown into childcare alone before fully recovering from childbirth	- First-time experience of overwhelming housework and the struggles of childcare
		- Studying, but finding no answers to childcare
	Receiving childcare information and comfort from fellow parents who share similar experiences	- The biggest source of quickly accessible childcare information
		- Only fellow parents understand the difficulties of childcare
	Always worried due to the lack of reliable information for the first child	- Lots of information, but hard to find reliable information
		- Need for personalized help from childcare experts for my child
Conflicted between being a stay-at-home mom and a working mom under inadequate maternity protection policies	Drifting away from society after childbirth	- Feeling socially isolated as conversations with my husband and coworkers shift after childbirth
		- Concerned about the possibility of returning to work while leaving my child behind
	Feeling like I should only have one child if I want to return to work	- Naturally letting go of my own needs after childbirth
		- One experience of self-sacrifice is enough for me
