# Corrigendum: Postnatal social support experiences in primiparous women in Korea: a phenomenological study

**DOI:** 10.4069/whn.2024.06.12.e1

**Published:** 2024-09-20

**Authors:** Eunjoo Lee, Kyongsuk Hong

**Affiliations:** Department of Nursing, Kyungnam University, Changwon, Korea

Womens Health Nurs 2024;30(2):140-152.

https://doi.org/10.4069/whn.2024.06.12

This corrigendum is issued to correct the Institutional Review Board (IRB) approval number that was mistakenly reported in the above article.

(Before correction) IRB-1040460-A-2020-039

(After correction) IRB-1040460-A-2022-033

The authors apologize for any inconvenience this may have caused.

